# Supplementary material for: Differences in PCV13 Recommendation Practices between Pediatric Care Providers and Primary Care Providers in China: A Cross-Sectional Survey of Behavior and Social Drivers
Source: Vaccines (Basel). 2024 Sep 22;12(9):1082. doi: 10.3390/vaccines12091082 (PMC11435928; doi:10.3390/vaccines12091082)
Supplement: Supplementary file 1 [file vaccines-12-01082-s001.zip › vaccines-3165206-supplementary.pdf]

Supplementary Table 1: Collinearity diagnostics.

	Pediatric care providers' s model		Primary care providers' s model	
	Tolerance values	VIF	Tolerance values	VIF
Vaccine training history in the past two years	0.727	1.376	0.762	1.312
Weekly work time	0.867	1.154	0.869	1.15
Willing to get influenza vaccine in the future	0.765	1.307	0.816	1.225
Average basic salary	0.67	1.493	0.713	1.402
Frequent inquiries about the vaccine	0.675	1.482	0.718	1.394
Familiar with PCV13 vaccination procedures	0.672	1.489	0.622	1.607
Understanding that children have a healthy lifestyle does not mean that they will be free from pneumonia, and there is still a need to vaccinate against pneumonia	0.748	1.338	0.807	1.239
Knowing that the newly marketed PCV13 does not carry a higher risk than PCV7	0.782	1.279	0.808	1.237
Understanding that if a child is not vaccinated against pneumonia, it increases the risk of pneumonia for the entire population	0.924	1.082	0.921	1.086
Perceived high susceptibility to pneumonia and the necessity of vaccination	0.253	3.952	0.251	3.981
Thinking that recommending PCV to parents would be undesireable	0.718	1.394	0.767	1.304
Thinking that the main reason why parents are reluctant to vaccinate their children against pneumonia is the high price	0.692	1.444	0.778	1.285
Agree that parents of children with better family conditions were more likely to accept the pneumonia vaccine recommendation	0.819	1.221	0.869	1.151
Agree that it is the responsibility of medical personnel to provide information and advice on pneumonia vaccinations to parents of children	0.81	1.234	0.865	1.156
Agree that their busy schedule has prevented them from introducing the pneumonia vaccine to the parents	0.64	1.562	0.664	1.506

Note-1: Variance inflation factor = $1/(1-R^2)$; Tolerance values=$(1-R^2)$

Note-2: The variables with Variance inflation factor >5 and the tolerance values greater than 0.1 will be regarded as collinear variables and cannot be included in the multiple regression model.
